# Evolutionary drivers of sex-specific parasite prevalence in wild birds

**DOI:** 10.1098/rspb.2024.1013

**Published:** 2024-08-07

**Authors:** José O. Valdebenito, William Jones, Tamás Székely

**Affiliations:** ^1^ Debrecen Biodiversity Research Centre, University of Debrecen, Debrecen, Hungary; ^2^ Bird Ecology Lab, Instituto de Ciencias Marinas y Limnológicas, Universidad Austral de Chile, Valdivia, Los Ríos, Chile; ^3^ Centro de Humedales Río Cruces (CEHUM), Universidad Austral de Chile, Valdivia, Los Ríos, Chile; ^4^ Millennium Institute Biodiversity of Antarctic and Subantarctic Ecosystems (BASE), Chile; ^5^ Milner Centre for Evolution, Department of Biology and Biochemistry, University of Bath, Bath, UK

**Keywords:** host–parasite, life-history, malaria, sexual dimorphism, sex roles, seasonality

## Abstract

Males and females often differ in ecology, behaviour and lifestyle, and these differences are expected to lead to sex differences in parasite susceptibility. However, neither the sex differences in parasite prevalence, nor their ecological and evolutionary drivers have been investigated across a broad range of taxa using phylogenetically corrected analyses. Using the most extensive dataset yet that includes 755 prevalence estimates from 151 wild bird species in a meta-analytic framework, here we compare sex differences in blood and gastrointestinal parasites. We show that despite sex differences in parasite infection being frequently reported in the literature, only *Haemoproteus* infections were more prevalent in females than in males. Notably, only seasonality was strongly associated with the sex-specific parasite prevalence of both *Leucocytozoon* and *Haemoproteus*, where birds showed greater female bias in prevalence during breeding periods compared to the non-breeding period. No other ecological or sexual selection variables were associated with sex-specific prevalence of parasite prevalence. We suggest that much of the variation in sex-biased prevalence could be idiosyncratic, and driven by local ecology and behavioural differences of the parasite and the host. Therefore, breeding ecology and sexual selection may only have a modest influence on sex-different parasite prevalence across wild birds.

## 1. Introduction

Differences in lifestyle between males and females may result in varying rates of exposure to pathogens, potentially leading to sex differences in pathogen burdens in natural populations [1–3]. Parasites play a crucial role as they can influence behaviour and population dynamics, including survival [4–6]. Consequently, the differential parasite burden between sexes could have significant consequences for a species.

In general terms, ecological variables such as population density, latitude, seasonality, nest type and location are examples of good predictors of different metrics of parasite infection in animals, including in birds [7–11]. Unfortunately, these general predictors usually have limited application when explaining sex-specific infections, though exceptions exist. For example, nest type might play a role if in association with parental duties of males and females at the nest, particularly because the sex that provides most of the pre-hatching parental care (i.e. incubating the eggs) will have an increased risk of vector attacks and therefore higher blood parasite infections rates [12,13]; but see [14]. Seasonality—differences between two or more periods within a bird's year cycle—also has shown evidence of determining parasite burden between the sexes, presumably mediated by immunological costs associated with, for example, courting, egg-laying and parental care, that could ultimately hamper defence against new infections or trigger relapses of latent infections [15–20].

Body size may become relevant when the size differences between the sexes (i.e. sexual size dimorphism, SSD) are considered, because it has been argued that the larger sex may have higher parasite diversity and load due to its greater physical space, higher food consumption that leads to greater exposure to gastrointestinal parasites, and because it might be more effective at attracting parasite vectors [10,21–23]. Moreover, SSD also has evolutionary support for sex differences in parasite burden. It has been hypothesized that the larger sex (typically males) would gain more if investing in mate attraction than in longevity (i.e. immune defence [24]). This is assumed to be true for most traits associated with sexual selection, such as sexual dichromatism (SDC) and mating system [25,26]. Meanwhile, despite parental care and sexual selection having good theoretical support for determining sex-specific parasite infection, few studies have previously addressed this subject in a comparative framework using wild bird species. One notable exception is an early study by McCurdy *et al*. [27], finding a female-biased infection in blood parasites when polygyny was present in breeding wild birds. Taken together, despite the well-acknowledged commonality of sex-specific parasite infection in wild birds, current research has not yet been able to determine consistent drivers of sex biases in parasite infection in these hosts.

Here we used phylogenetic comparative methods to explore the predictors of sex-specific parasite prevalence using meta-analytic methods in adult birds from the wild. Our analysis used data collected from the literature on parasite prevalence from males and females of 151 avian hosts (108 for blood parasites and 60 for gut parasites), including blood parasites of the genera *Haemoproteus* (including *Parahaemoproteus*), *Leucocytozoon*, *Plasmodium* and *Trypanosoma*; and gut parasites of the groups Acanthocephala, Cestoda, Nematoda, Trematoda and Protozoans. First, we determined both mean sex difference in parasite prevalence (standardized mean difference, Hedges' *d*) and sex difference in parasite variance (natural logarithm of coefficient of variation, lnCVR) to characterize sex differences in parasitism. Early research suggests a possible male bias in some groups of gut parasites, though more recent comparative studies incorporating sex steroids, sex-specific behaviour, immunity and parasitism suggest little to no overall sex difference in parasite prevalence in both gut and blood parasites in birds [20,28–31]. The analysis was complemented by exploring whether closely related host species had similar levels of sex-specific parasite prevalence [32] by determining the phylogenetic signal of each parasite type (Pagel's *λ* and Blomberg's *K* [33,34]). Second, we tested seven predictors thought to be relevant for sex-specific parasite infection under different hypotheses: seasonality, nest type, incubation, chick feeding, SSD, SDC and mating system. We tested Hedges' *d* and the lnCVR of blood parasites against all seven predictors, but gut parasites were only tested against sexual selection predictors since sex-specific exposure to gut parasites does not seem to relate to parental provisioning [35,36]. In the analysis of seasonality, we expected a female bias in parasite prevalence in the breeding season compared to the non-breeding season, due to the possible unfavourable effects associated with reproduction in females over males [37]. Assuming that the majority of parental care is provided by females (e.g. sitting in the nest during incubation [38]), we expected higher blood parasite prevalence in females than males due to their differential exposure to vectors, in addition to possible relapses of latent infections. On the contrary, because males usually have the highest variance in reproductive success (typically the competing sex), we predicted that their prevalence of blood and gut parasites would surpass female prevalence [5].

## 2. Methods

### (a) Data collection

We used the sex-specific parasite prevalence data published by Valdebenito *et al*. [29] that covered until 2017. We then augmented their data up until May 2021 by systematically collecting data on sex-specific blood and gut parasites from birds (PRISMA method [39]) using ISI Web of Science (see chart in electronic supplementary material, file 1 and figure S1; full list of references during the different filtering steps provided in electronic supplementary material, file 2). Our inclusion criteria required these data to be: (i) determined from adult birds with known sex (molecular or morphological sexing), (ii) obtained from free-living wild birds (not captive), (iii) from populations that were not experimentally manipulated, (iv) reported for both males and females from the same population of origin and sampled both within the same timeframe. In order to conduct the meta-analytic calculations, the selected studies should have presented their results in a way that allowed determining the number of examined and infected birds per sex. Altogether, 150 publications qualified to be included in the analysis, extending across 151 species (108 for blood parasites, 60 for gut parasites, 17 species for which we had blood and gut parasite data) of 46 avian families (electronic supplementary material, table S1).

### (b) Estimating sex bias in parasite infection (effect sizes)

Sex bias in parasite prevalence was estimated as the standardized mean difference between male and female parasite prevalence, using Hedges’ *d* as effect size. Hedges' *d* has been widely used in ecology and evolution studies (sometimes referred as Hedges’ *g*) due to its bias correction for small sample sizes. We calculated Hedges' *d* and its sampling variance following Hedges & Olkin [40] and Cohen [41]. To quantify sex differences in the variance in parasite prevalence (hereafter parasite variability) we used lnCVR, proposed by Nakagawa *et al*. [42]. For Hedges’ *d* and lnCVR, positive values represent a male bias and negative values a female bias. The advantage of using effect sizes is that, unlike sex differences in average values, it considers the variance within sex and provides a value that reflects both the direction and magnitude of the sex difference in parasite prevalence, while being independent of the scale of the original variable.

### (c) Explanatory variables

Data on breeding ecology, sex roles and sexual selection were extracted from Gonzalez-Voyer *et al*. [26] and Storchova & Horak [43]. Missing data were completed, to our best effort, by consulting various ornithological literature sources, in particular, Birds of the World (The Cornell Ornithology Lab [44]), and local experts.

#### (i) Seasonality

Two-level categorical variable: ‘breeding’, ‘non-breeding’. At the moment of parasite data collection, we noted the period within the bird's year cycle where sampling took place. We considered a population as breeding if parasite determination was estimated at any point within the very beginning of the breeding season, including for example territory determination, until the end of chick feeding. This period usually takes place in spring and summer, or during the rainy season in tropical birds. In most cases the sampling period was stated; however, when this information was missing, we inferred it in different manners. For instance, birds were considered breeding if sampled at the nest, or if sampling dates fell well within the described breeding period for a species/population (The Cornell Ornithology Lab [44]). We also contacted authors to request missing breeding status information. Everything sampled in a time period outside what was described above was categorized as non-breeding. A third category that included studies reporting results combined for both breeding and non-breeding seasons was also noted but excluded from the analysis.

#### (ii) Nest type

Two-level categorical variable: ‘open-cup’, ‘closed-cup’. These were obtained based on the Lutz *et al*. [9] nest type qualification system, but instead of dividing bird nests into three types (open-cup, closed-cup and cavity nesting), we merged cavity nesting and closed-cup into one: closed-cup.

#### (iii) Incubation and chick feeding

These variables corresponded to a 5-point score of the relative effort of the male, where: 0 = no involvement of male; 1 = 1–33%; 2 = 34–66%; 3 = 67–99%; 4 = 100%.

#### (iv) Sexual size dimorphism

We used this variable as a proxy for mate competition (sexual selection), and it was computed as log(adult male body mass (g)/adult female body mass (g)).

#### (v) Sexual dichromatism

We used the plumage scoring system provided by Gonzalez-Voyer *et al*. [26] (which contains full details). In short, the bird was split into five body regions (head; nape, back and rump; throat, chest and belly; tail; and wings). For a given species, each body region was scored separately with a score system that ranged between –2 and 2, according to the following: –2, the female was substantially brighter and/or more patterned than the male; –1, the female was brighter and/or more patterned than the male; 0, there was no difference in the body region or there was difference but neither could be considered brighter than the other; 1, the male was brighter and/or more patterned than the female; 2, the male was substantially brighter and/or more patterned than the female. For the analysis we used the median of these five regions.

#### (vi) Mating system

We used the difference between scores of female polygamy and male polygamy as a proxy for the social mating system based on the scoring system in Liker *et al*. [38]. The overall incidence of polygamy for each sex was expressed on a 5-point score, with 0 corresponding to no (or very rare) polygamy (less than 0.1% of individuals), 1 to rare polygamy (0.1–1%), 2 to uncommon polygamy (1–5%), 3 to moderate polygamy (5–20%), and 4 to common polygamy (greater than 20%; including males in lekking species to express the high variance in male mating success in these species [45]). We calculated the sex difference in social mating system as male score minus female score. Further details are in the electronic supplementary material.

### (d) Statistical analysis

Meta-analyses: we modelled the effect sizes Hedges' *d* and lnCVR using multi-level meta-analytic (MLMA) models (intercept-only models that consider random effects) and then ran multi-level meta-regression (MLMR) models (including fixed-effect moderators) in R version 4.3.3 [46] using the package *metafor* version 4.6-0 [47]. Phylogeny (a variance–covariance matrix), species (to correct for species for which we had several estimates) and study (to account for more than one species and/or parasite estimate per study) were added as random-effect variables. We used the avian phylogeny proposed by Jetz *et al*. [48], using consensus trees obtained by 50% majority-rule [49,50] from 1000 randomly selected trees from a pool of 10 000 available trees (http://birdtree.org) using the methodology described by Rubolini *et al*. [51]. These phylogenetic trees were not fully resolved, and polytomies were arbitrarily resolved by adding a branch distance of 10^–8^ to one randomly chosen branch in the polytomy using the function *multi2di* from the R package *ape* [52]. We also calculated the heterogeneity due to phylogenetic relatedness ($I_{\mathrm{phy}}^{2}$), differences among studies ($I_{\mathrm{study}}^{2}$), differences between species ($I_{\mathrm{species}}^{2}$) and the total variance attributed to the random effect variables (i.e. sum of the three effects, $I_{\mathrm{total}}^{2}$). Orchard plots were generated using the recommendations of Nakagawa *et al*. [53].

Different MLMR models were run for Hedges’ *d* and lnCVR effect sizes. Breeding ecology and sex roles variables (seasonality, nest type, incubation and chick feeding) were used only in blood parasite models as multi-predictor (or moderator) models, with the inclusion of a two-way interaction between incubation and nest type. The analysis of seasonality was conducted with a subset of the data, only including those species for which we had both breeding and non-breeding data available. Only *Leucocytozoon*, *Haemoproteus* and *Plasmodium* met these criteria. These models had seasonality as the only fixed predictor. Because *Trypanosoma* models had a low sample size, we only included two predictors for this parasite: incubation and chick feeding. Theory says that variables related to sexual selection such as SSD, SDC and mating system would strongly correlate to each other; however, a recent comparative study showed that in birds, these variables were not as strongly correlated as previously thought [26]. We ran Spearman's rank correlation between the three variables, showing that indeed the correlation was weak, with the highest being between mating system and SSD (mating system and SSD, *ρ* = 0.146, *p*-value = 0.047; mating system and SDC, *ρ* = 0.127, *p*-value = 0.083; SSD and SDC, *ρ* = 0.009, *p*-value = 0.899). In this case, we opted to conduct the analysis of sexual selection variables using the most cautious approach, i.e. running single predictor meta-regression models with one variable at a time. Models of gut parasites were run only with sexual selection variables whereas blood parasites modes were run for all variable types. Acanthocephalans and Protozoans were excluded from MLMR models to avoid over-parametrization due to their low sample sizes.

Publication bias: this represents a threat to the validity of quantitative evidence in meta-analyses as it results in some findings being overrepresented in meta-analytic datasets because they are published more frequently or sooner (e.g. statistically significant results [54]). We used the ‘all-in publication bias test’ method, suggested by Nakagawa *et al*. [55], which consists of adding the sampling error (of the effect sizes) along with the other moderators. This method is a variant of the Egger regression test [56]. We determined Cook's distance [57], ‘hat values’ and evaluated funnel plots to identify influential estimates responsible for the publication bias. We found partial evidence of publication bias which was more prevalent in Hedges' *d* than in lnCVR estimates. Further details are provided in the electronic supplementary material.

Phylogenetic signal: we calculated phylogenetic signal for host species applying the methods of Pagel's *λ* [33] and Blomberg's *K* [34], using the *phylosig* function in the R package *phytools* [58]. This analysis was conducted only in parasite groups with a sample size larger than 20 [59].

We excluded species where studies reported zero prevalence of blood parasites for both sexes (*n* = 30; mostly shorebirds and seabirds) since it raises the uncertainty of whether those individuals were able to clear the infection or simply never got infected. Nevertheless, we also provide results including these species in the electronic supplementary material. The main analysis for blood parasites included 108 species and 36 families, whereas the supplementary analysis had 138 species and 47 avian families. Both main and supplementary blood parasites analyses yielded largely similar outcomes (see electronic supplementary material, tables S2 and S3), which shows the robustness of our analysis. *p*-values were corrected for multiple testing [60].

## 3. Results

### (a) Phylogenetic distribution of parasites

**Figure 1.**
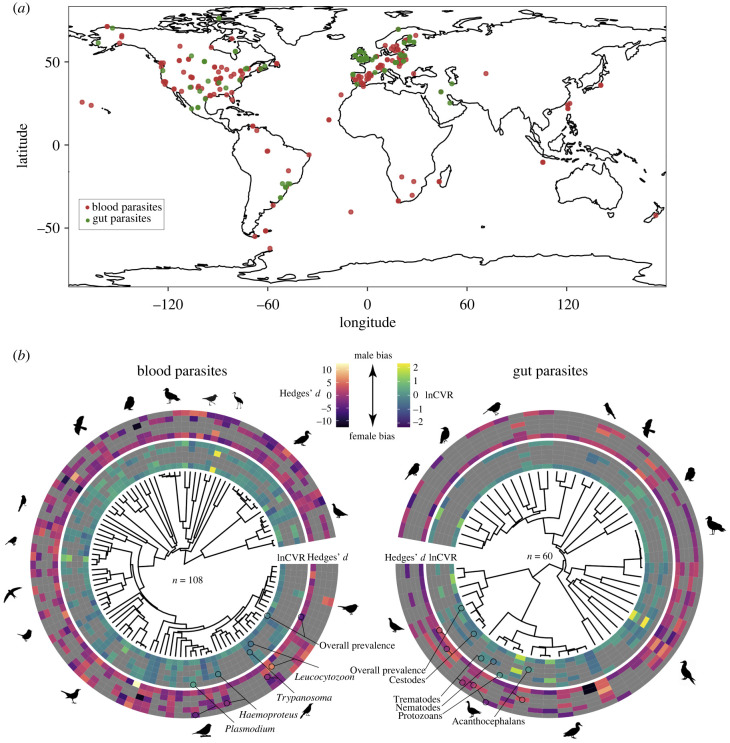
Global distribution of sex bias in parasite prevalence and parasite variability in 151 bird species representing 16 avian orders. (*a*) Geographical distribution of sex-specific parasite data representing 474 and 281 estimates for blood and gut parasites, respectively, extracted from 150 studies. (*b*) Sex biases in parasite prevalence and parasite variability across 108 and 60 bird host species parasitized with blood and gut parasites, respectively. Outer rings show the sex bias in parasite prevalence (Hedges' *d*) and inner rings the sex bias in parasite variability (lnCVR). Grey squares indicate lack of data for a particular host species and parasite type.

Our final dataset comprised 755 effect sizes across 151 avian host species (electronic supplementary material, table S1), for which we had sex-specific data of at least one of the following parasite types: blood parasites: *Haemoproteus*, *Leucocytozoon*, *Plasmodium*, *Trypanosoma*; and gut parasites: Acanthocephala, Cestoda, Nematoda, Trematoda and Protozoans (sample sizes in table 1). Studies with the largest sample sizes in blood parasites investigated hosts such as the black-capped chickadee (*Parus atricapillus*; *n* = 1045 ♀ and 991 ♂), whereas in gut parasites the European greenfinch (*Chloris chloris*; 391 ♀ and 490 ♂). The smallest sample sizes were reported from white-necked thrush (*Turdus albicollis*; 4 ♀ and 5 ♂) and the northern fulmar (*Fulmarus glacialis*; 7 ♀ and 9 ♂) in blood and gut parasites, respectively. The data coverage was global, although Europe and North America provided more data than other geographical areas (figure 1).

**Table 1.** Multi-level meta-analysis (MLMA) model summaries for blood and gut parasites. We report the standardized mean difference (and 95% confidence intervals, CI) between the sexes (Hedges' *d*) and ln coefficient of variation (lnCVR). Number of effect sizes (*k*), species (*sp*) or studies (*st*) may differ depending on the publication bias test outcomes of each model. Total heterogeneity ($I_{\mathrm{total}}^{2}$) corresponds to $I_{\mathrm{phy}}^{2}+I_{\mathrm{study}}^{2}+I_{\mathrm{species}}^{2}$. Phylogenetic signal was calculated for Pagel's *λ* and Blomberg's *K*, with their corresponding *p*-values in parentheses. Estimates with *p-*values < 0.05 are highlighted in bold.

	mean	95% CI	*p*-value	*k*	*sp*	*st*	$I_{\mathrm{total}}^{2}$	$I_{\mathrm{phy}}^{2}$	$I_{\mathrm{study}}^{2}$	$I_{\mathrm{species}}^{2}$	*λ*	*K*
blood parasites												
Hedges’ *d*												
*Leucocytozoon*	0.320	−0.477, 1.117	0.852	61	40	37	99.2	0	29.2	70.0	0 (>0.9)	0.22 (0.15)
*Trypanosoma*	0.524	−4.963, 6.01	0.852	17	16	17	99.8	85.3	14.5	0	**1** **(0.032)**	**0.82** **(0.006)**
*Haemoproteus*	**−1.680**	**−2.691, −0.669**	**0.008**	**84**	**62**	**56**	99.5	0.001	63.3	36.2	0 (>0.9)	0.08 (0.84)
*Plasmodium*	−0.442	−1.560, 0.677	0.852	70	49	42	99.7	0.005	38.4	61.3	0.01 (>0.9)	0.16 (0.21)
lnCVR												
*Leucocytozoon*	−0.041	−0.297, 0.216	0.852	62	40	37	88.1	56.8	14.5	16.9	0.27 (0.489)	0.25 (0.091)
*Trypanosoma*	−0.138	−0.355, 0.079	0.852	21	16	17	84.6	27.6	0	57.1	0.18 (>0.9)	0.39 (0.229)
*Haemoproteus*	−0.023	−0.147, 0.102	0.852	86	62	57	75.8	25.9	41.7	8.1	0.09 (0.551)	0.13 (0.487)
*Plasmodium*	−0.033	−0.189, 0.124	0.852	73	49	42	84.6	31.4	46.8	6.5	0 (>0.9)	0.16 (0.257)
gut parasites												
Hedges' *d*												
Cestodes	0.351	−0.962, 1.664	0.750	25	23	21	99.2	0	53.43	45.7	0 (>0.9)	0.14 (0.38)
Acanthocephalans	0.892	−0.911, 2.695	0.593	13	12	11	99.4	0	99.35	0		
Nematodes	0.883	−0.510, 2.277	0.593	38	28	24	99.4	0	45.48	53.9	0 (>0.9)	0.21 (0.07)
Trematodes	−0.183	−2.223, 1.856	0.860	42	24	16	98.8	38.3	35.75	24.8	0 (>0.9)	0.06 (0.19)
Protozoans	−2.170	−4.059, −0.281	0.240	17	17	7	99.4	0	73.6	25.8		
lnCVR												
Cestodes	−0.077	−0.238, 0.083	0.593	28	24	22	90.8	0.8	68.36	21.6	0 (>0.9)	0.17 (0.19)
Acanthocephalans	−0.059	−0.241, 0.124	0.750	15	12	11	79	0.87	72.66	5.5		
Nematodes	−0.127	−0.265, 0.012	0.370	38	26	23	81.9	0	70.86	11.04	0 (>0.9)	0.13 (0.5)
Trematodes	−0.032	−0.206, 0.143	0.801	46	24	16	88.1	0	25.14	63.0	0 (>0.9)	0.03 (0.74)
Protozoans	0.173	−0.194, 0.539	0.593	18	18	7	93.4	9.46	83.95	0		

Only *Trypanosoma* parasites showed evidence of phylogenetic signal, where both Pagel's *λ* and Blomberg's *K* were statistically significant (table 1), suggesting that closely related host species exhibit similar sex bias in prevalence of this blood parasite. No other parasite group showed evidence of phylogenetically conserved parasite prevalence among closely related host species.

### (b) Sex bias in parasite prevalence

**Figure 2.**
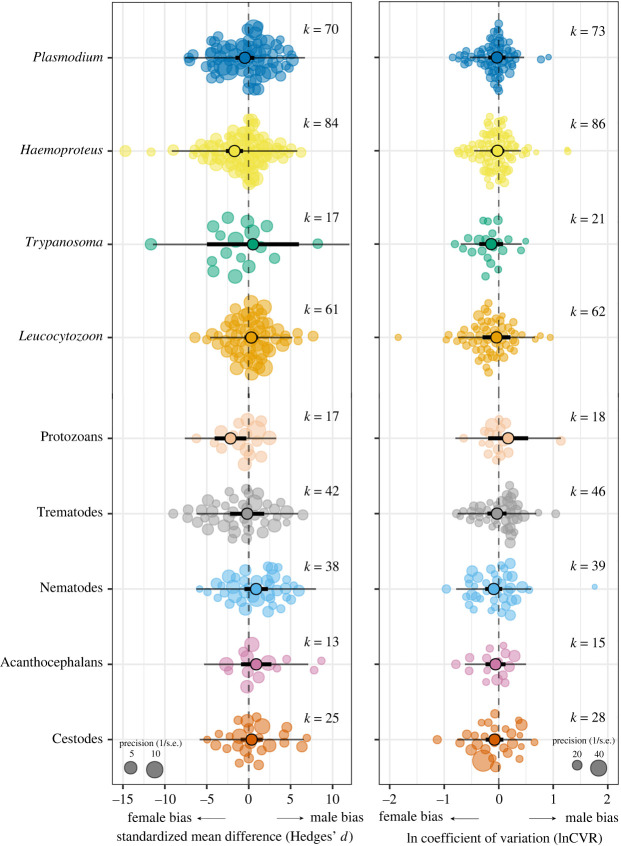
Sex bias in blood and gut parasite prevalence in adult wild birds. Orchard plot of sex differences in mean parasite prevalence (Hedges’ *d*) and parasite variability (lnCVR) in blood and gut parasites. Positive estimates represent a male bias and negative a female bias. Thick bars are upper and lower 95% confidence intervals and thin bars are prediction intervals. Circle size reflects effect size precision (inverse of standard error). *k* = number of effect sizes. Full results in table 1.

Across the nine parasite groups, we found that the standardized mean difference (Hedges' *d*) between sexes of *Haemoproteus* was significantly biased towards females (table 1 and figure 2). None of the other parasite groups showed significant sex bias, though there was substantial variation in sex biases across parasite types (table 1 and figure 2).

Heterogeneity was high in all models, with total heterogeneity ($I_{\mathrm{total}}^{2}$) ranging from 75.8 to 99.8% (table 1). In blood parasite models, the three random variables—phylogeny, species and study ID—explained the variation rather equally. In gut parasite models, phylogeny explained little to no variation, whereas species and study ID explained almost all of the variation (table 1).

### (c) Predictors of sex-specific parasite infection

**Figure 3.**
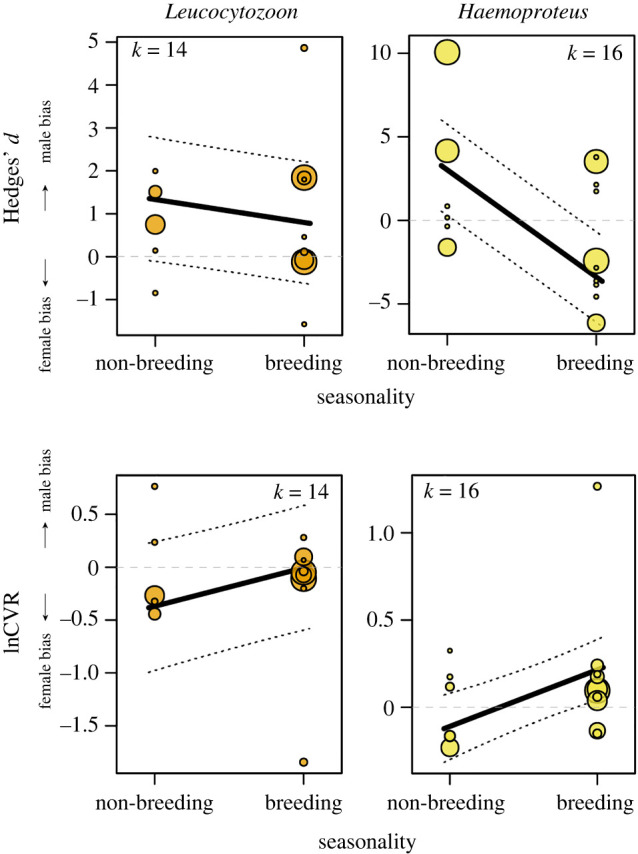
Sex bias in blood parasitism in association with seasonality. Multi-level meta-regression (MLMR) of the sex bias in parasite prevalence (top row, Hedges' *d*) and parasite variability (bottom row, lnCVR) in blood parasites. This analysis only included species for which there were available both breeding and non-breeding data. Regression lines computed from corresponding models. Circle size reflects effect size precision (inverse of standard error). *k* = number of effect sizes. Full results presented in table 2.

Across our model testing, only seasonality was associated with sex-specific blood parasite infection (table 2), where Hedges’ *d* of both *Leucocytozoon* and *Haemoproteus* had a negative association with seasonality, meaning that parasite prevalence becomes more female-biased when it transitions from non-breeding to the breeding season (table 2 and figure 3). Conversely, lnCVR models showed that parasite variability was more male-biased during the breeding season compared to the non-breeding season (table 2 and figure 3). None of the other sexual selection or ecological variables showed an association with sex-specific parasite infection (full results in electronic supplementary material, table S4).

**Table 2.** Multi-level meta-regression (MLMR) model summaries of seasonality in blood parasites. The analysis was conducted with a subset of species for which there were parasite prevalence estimates for both the breeding and non-breeding seasons. We report the standardized mean difference (Hedges' *d*) and ln coefficient of variation (lnCVR). Number of effect sizes (*k*), species (*sp*) or studies (*st*) may differ depending on the publication bias test outcomes of each model. Estimates with *p*-values < 0.05 are highlighted in bold.

	Hedges' *d*						lnCVR					
	mean	95% CI	*p*-value	*k*	*sp*	*st*	mean	95% CI	*p*-value	*k*	*sp*	*st*
*Leucocytozoon*												
season (breeding)^a^	**−0.535**	**−0.894, −0.177**	**0.044**	**14**	**4**	**8**	**0.363**	**0.145, 0.581**	**0.019**	**14**	**4**	**8**
*Haemoproteus*												
season (breeding)^a^	**−6.411**	**−7.043, −5.779**	**<0.001**	**16**	**6**	**12**	**0.324**	**0.171, 0.478**	**<0.001**	**16**	**6**	**12**
*Plasmodium*												
season (breeding)^a^	−0398	−0.984, 0187	0.505	11	4	9	0.141	−0.044, 0.326	0.464	13	4	9

^a^Relative to the non-breeding season.

## 4. Discussion

This work represents the first comprehensive study of evolutionary and behavioural causes of parasite infection from a sex-specific perspective, including both blood and gut parasites, across wild bird species. Our study shows that the sex differences in parasite prevalence and variability were scarce among birds, restricted only to a female-biased infection in *Haemoproteus* parasites. Interestingly, the sex bias in parasite infection was broadly unrelated to most of the evolutionary and ecological variables here tested, although some parasite groups such as *Leucocytozoon* and *Haemoproteus* seem to be importantly determined by seasonal variations in the sexes.

Data coverage showed good global representation though specific areas could benefit from greater presence, such as Australia, East Asia and the tropics. Unsurprisingly, most data originated from North America and Europe. The tropics represent an unfortunate gap because they host the greatest coloration and dimorphism among wild birds [61], which is relevant for testing hypotheses that relate to sexual selection theory (see below).

As is frequent in meta-analyses in ecology and evolution, heterogeneity in our study was high, and an important proportion of the variation was explained by species and study ID which is thought to emerge due to the system-specific nature of biological phenomena [62].

The phylogenetic signal among hosts was absent except for *Trypanosoma* infection (both Blomberg's *K* and Pagel's *λ*). This result was also supported by the heterogeneity of phylogeny ($I_{\mathrm{phy}}^{2}$) of 85% in *Trypanosoma*. This suggests that either the defence mechanisms (of the host), or the means of infection of *Trypanosoma* are phylogenetically conserved between the sexes [63]. In two previous studies, not considering sex, only *Leucocytozoon* infection but not *Haemoproteus* and *Plasmodium* showed significant phylogenetic signal in Scandinavian passerines [32], and *Haemoproteus* infection had higher phylogenetic signal than *Plasmodium* among tropical bird communities in south India [64].

The first part of the analysis shows a female-skew in prevalence of *Haemoproteus*. Our results support the work of McCurdy *et al*. [27] that only found a female-biased *Haemoproteus* infection, though the present study surpasses their sample size nearly six-fold. On the other hand, a previous meta-analysis on gastrointestinal parasites by Poulin [1] found that males had higher parasite prevalence than females in Acanthocephala and Cestoda parasites across six wild bird hosts. We found no such sex biases, though differences in sample sizes and methodologies are likely sources of discrepancies between the results. The reasons for a female-biased prevalence could be numerous. One main driver could be greater exposure to the most frequent insect vectors, either louse flies (Hippoboscidae) for the subgenera *Haemoproteus* or biting midgets (genus *Culicoides*) for *Parahaemoproteus* parasites. Therefore, if females spend more time at the nest or have weaker/more permissive immune defences, this could translate into higher parasite infection rates [65]. Some evidence supports this since a previous comparative study found a male bias in several immune parameters during the transition from the non-breeding to the breeding period in wild birds [20]. None of the other eight parasite groups showed statistically significant sex differences. This is in accordance with previous studies using modern meta-analytic methods showing, in general, marginal to small sex differences across a number of traits in animals [28,29,31]. For instance, Harrison *et al*. [30] explored sex differences in personality traits including sociability, exploration and activity (time spent being active). These traits could be associated with aspects of parasite acquisition since, for example, more time spent being active and exploring could entail higher chances of encountering gut parasites via food consumption. However, the sex differences in these behavioural traits in birds were little to none [30].

Across all meta-regression models, there was no consistent predictor of sex-specific prevalence. This suggests that, despite plausible theoretical support for some proposed associations (see Introduction), parasite prevalence of males and females across wild birds is independent of sexual selection and most ecological variables. The lack of association found here could relate to the fact that the infection of the host is often the result of a complex chain of events, which may include environmental conditions, intermediate hosts and vectors, and the immune status of the avian host. We hypothesize that components yet unknown, but specific to either gut or blood parasites, could mediate the pattern. For instance, there is intriguing experimental evidence suggesting that vectors may prefer one sex over the other in some circumstances (see [66]); however, more work is needed to explore how common this trend is in nature. *Leucocytozoon* and *Haemoproteus* showed a sex-specific seasonal pattern, where parasite prevalence was more female-biased during the breeding season than during the non-breeding season. There is a common trend for host populations to exhibit higher parasite prevalence during the summer or the wet season (i.e. the breeding season for most birds) and that infections are higher in females than males (e.g. [7,67,68]). This could be explained by several non-exclusive mechanisms, for example higher infection risk for females, or that females are less efficient at clearing or suppressing infections than males. It is interesting that recent evidence supports the latter case, since Valdebenito *et al*. [20] found that females but not males showed reduced levels of some immune parameters during the breeding season compared to the non-breeding season. However, we note that these results should be taken cautiously because the number of effect sizes and host species was rather small. If the seasonal variation in immunocompetences were true, it also opens the possibility of relapses of previous latent infections, that become patent once the bird starts to undergo immunosuppression [15,17]. Alternatively, perhaps there could be sex-specific trade-offs between resistance and tolerance, where males opt to resist and clear parasites, while females are more tolerant to infections [69–71]. However, this research topic needs further exploration.

Sex differences in parasite variability and its association with seasonality can be challenging to interpret. One possible explanation could emerge from the diversity of sex roles between males and females in birds. Considering that in birds the female would most certainly contribute with at least half of the duties during breeding, in addition to egg production, it could be expected that the variation in infection rate is much more homogeneous that the one of the male, that at most, would contribute with half of the duties [26,72]. These ideas could therefore be used to explain why parasite variability became more male-biased when transitioning from non-breeding towards the breeding season.

In conclusion, despite sex differences in parasite prevalence emerging frequently among specific wild bird species, these sex differences seem to be much less apparent when investigating across birds. From nine parasite groups investigated, only *Haemoproteus* showed a female bias in prevalence. One important evolutionary implication is that parasitism could relate to mortality in animals, including in birds [73]. Considering that *Haemoproteus* parasites are thought to mostly cause sublethal damage to the host, we could argue that it is unlikely that a sex-biased infection could importantly influence mortality in wild birds [29]. Despite having consistent associations between some sex role and sexual selection variables and parasitism, most of the associations we found were rather statistically weak. This suggests that the main drivers of sex-specific parasite prevalence are still unknown, perhaps because much of the variation could be driven by local ecology and behavioural differences in male and female lifestyles. We hope future studies with additional data will advance this enigmatic topic. For example, we acknowledge that blood parasites are particularly challenging to put into a sex-specific perspective because infection not only depends on the host's defence but also vector availability and biting preferences. Perhaps an important limitation to highlight in our work is that studies investigating sexually dimorphic birds are more likely to report results separated by sex, though this is expected to be, at least partly, corrected once publication bias was addressed. Lastly, we note and welcome an increased awareness and reporting of parasitology studies that explicitly incorporate sex into their studies, which is critical to develop our understanding of the role of sex in biological processes.

## Data Availability

Data and code available from Figshare [74]. Electronic supplementary material is available online [75].
